# Proinflammatory Cytokine Gene Expression by Murine Macrophages in Response to *Brugia malayi Wolbachia* Surface Protein

**DOI:** 10.1155/2007/84318

**Published:** 2007-05-03

**Authors:** Chantima Porksakorn, Surang Nuchprayoon, Kiwon Park, Alan L. Scott

**Affiliations:** ^1^Lymphatic Filariasis Research Unit, Department of Parasitology, and Chulalongkorn Medical Research Center, Faculty of Medicine, Chulalongkorn University, Bangkok 10330, Thailand; ^2^Department of Molecular Microbiology and Immunology, Bloomberg School of Public Health, Johns Hopkins University, Baltimore, MD 21205, USA

## Abstract

*Wolbachia*, an endosymbiotic bacterium found in most species of filarial parasites, is thought to play a significant role in inducing innate inflammatory responses in lymphatic filariasis patients. However, the *Wolbachia*-derived molecules that are recognized by the innate immune system have not yet been identified. In this study, we exposed the murine macrophage cell line RAW 264.7 to a recombinant form of the major *Wolbachia* surface protein (rWSP) to determine if WSP is capable of innately inducing cytokine transcription. Interleukin (IL)-1*β*, IL-6, and tumor necrosis factor (TNF) mRNAs were all upregulated by the rWSP stimulation in a dose-dependant manner. TNF transcription peaked at 3 hours, whereas IL-1*β* and IL-6 transcription peaked at 6 hours post-rWSP exposure. The levels of innate cytokine expression induced by a high-dose (9.0 *μ*g/mL) rWSP in the RAW 264.7 cells were comparable to the levels induced by 0.1 *μ*g/mL *E. coli*-derived lipopolysaccharides. Pretreatment of the rWSP with proteinase-K drastically reduced IL-1*β*, IL-6, and TNF transcription. However, the proinflammatory response was not inhibited by polymyxin B treatment. These results strongly suggest that the major *Wolbachia* surface protein molecule WSP is an important inducer of innate immune responses during filarial infections.

## 1. INTRODUCTION

Lymphatic filariasis remains a major debilitating and disfiguring disease that affects approximately 120 million people worldwide [1]. *Wuchereria bancrofti* is the major filarial species in most endemic areas, including Thailand [2–7]. The remaining cases of lymphatic filariasis are caused by *Brugia malayi* and *B. timori*. Host immune responses are thought to be a major factor contributing to disease progression in lymphatic filariasis, which manifests as either acute or chronic inflammation [8–10]. The adverse reactions associated with chemotherapeutic treatment are also thought to be due to inflammatory responses directly induced by molecules liberated from drug-damaged microfilariae [1, 11]. The drug-induced adverse reactions are associated with the increased post-treatment concentrations of proinflammatory cytokines and immune modulators, including tumor necrosis factor (TNF), interleukin (IL)-6, IL-10, lipopolysaccharide-binding protein (LBP), and soluble TNF receptors (sTNF-Rs) [12–14]. Although it is believed that innate immune responses play a major role in this immune-mediated pathology, the nature of the parasite-derived molecules that mediate this pathogenesis has not been defined.

A majority of filarial nematode species harbor an endosymbiotic bacterium from the genus *Wolbachia*. The results of genome sequence analysis [15, 16] and studies where the bacterium is cleared with antibacterial treatment suggests that the *Wolbachia* and the worm have established a mutualistic relationship in which *Wolbachia* appears to make major contributions to the developmental and reproductive biology of the nematode host [17, 18]. In addition, the *Wolbachia* from various filarial parasites have been implicated in the immunopathogenesis of filarial diseases [19–21]. In patients infected with *B. malayi*, the presence of *Wolbachia* following DEC treatment of parasites is strongly associated with severe systemic inflammatory reactions [22]. Initial studies to identify the molecular basis for bacteria-mediated inflammation suggested that *Wolbachia*-derived lipopolysaccharide (LPS) played a major role in inducing inflammatory responses [23]. However, a role for an LPS is unlikely since the *Wolbachia* genome does not contain the genes encoding for the enzymes required for the biosynthesis of LPS [15]. Therefore, other *Wolbachia*-derived molecules are responsible for LPS-like activity in worm extracts and these molecules might be important for the induction of adverse reactions associated with parasite death.

The purified recombinant form of the *Wolbachia* surface protein (rWSP) from *Wolbachia* of the dog heartworm *Dirofilaria immitis* elicits the secretion of IL-1*β*, IL-6, IL-8, and TNF from peripheral blood mononuclear cells (PBMC) of healthy people [24]. However, roles of WSP from *Wolbachia* from the human pathogen *B. malayi* in activating the innate immune system have not been characterized. The *B. malayi Wolbachia* WSP, a major component of the proteome [25], shares conserved regions with that of *Wolbachia* from other filarial parasites, and with outer membrane proteins of the closely related bacteria. In this study, we report that a recombinant form of the WSP from the *B. malayi* *Wolbachia* is a potent elicitor of the transcription of proinflammatory cytokines in murine macrophage cell line RAW 264.7.

## 2. MATERIALS AND METHODS

### 2.1. Cell culture

The murine macrophage cell line RAW 264.7 was purchased from the American Type Culture Collection (ATCC) and cultured in Dulbecco's modified Eagle's medium (DMEM; Gibco-BRL, Gaithersburg, Md, USA) containing 10% heat-inactivated fetal bovine serum in a humidified atmosphere of 5% CO_2_ and 95% air.

### 2.2. Cloning, expression, and purification of rWSP

The entire sequence of the gene encoding the *Wolbachia* surface protein minus the predicted N-terminal signal sequence was directionally cloned from genomic DNA extracted from *B. malayi* by polymerase chain reaction (PCR). The forward and reverse primers were 5′CACCATGGATCCTGTTGGTCCAATAGC3′ and 5′TTAGAAATTAAACGCTATTCCAGC3′, respectively. The gene was cloned into the pET100/D-TOPO expression vector (Invitrogen, Carlsbad, Calif, USA) and transformed into one-shot TOP10 cells (Invitrogen). Plasmids containing inserts were selected by amplicilin resistance and sequenced to confirm that the WSP gene was intact and in the correct orientation.

For expression, the WSP plasmid was transformed into *E. coli* BL21 (DE3) cells (Invitrogen). The rWSP-tagged fusion protein was then induced to express at 37°C with 1 mM isopropyl *β*-D-thiogalactopyranoside (IPTG) (Invitrogen). The rWSP was first purified by treatment of lysozyme and B-PER bacteria protein extraction reagent, according to the manufacturer's instructions (Pierce, Rockford, Ill, USA), and then affinity-purified by chromatography with Ni-NTA resin (Qiagen Inc., Valencia, Calif, USA). The purified rWSP protein was refolded upon dialysis. Protein concentration was determined by a bicinchoninic acid (BCA) protein assay (Pierce). The purity of the rWSP preparation, as determined by matrix-assisted laser desorption/ionization time of flight mass spectrometry analysis, was >90%. The rWSP preparation contained 0.17 EU/mL of endotoxin as determined by the *Limulus* Amebocyte Lysate test (BioWhittaker Inc., Walkersville, Md, USA; limit of detection of the assay was 0.06 EU/mL).

### 2.3. Treatments of murine macrophage RAW 264.7 cells with rWSP

1 × 10^5^ RAW 264.7 cells were plated into individual wells of 6-well plate and grown to ∼75% confluence at 37°C. The cells were exposed to concentrations of the rWSP that ranged from 9 to 0.1 *μ*g/mL. The macrophage cells were also stimulated with 0.1 *μ*g/mL *E. coli* LPS B026:B6 (Sigma, St. Louis, Mo, USA). For proteinase K-treatment, 100 *μ*g of the rWSP was treated with 1 mg of proteinase K at 55°C overnight, and inactivated at 95°C for 10 minutes. Cell cultures were treated with 10 *μ*g (80.7 U)/mL polymyxin B sulfate (Sigma). Immunoblotting with anti-rWSP antibodies was used to confirm the complete digestion of the rWSP. The cells were incubated with each treatment at 37°C for 3 hours, except for the time-course experiment.

### 2.4. Relative quantification of proinflammatory cytokine mRNAs by real-time RT-PCR

The RAW 264.7 cells were extracted for total RNA by using RNeasy mini kit (Qiagen Inc.), according to the manufacturer's instructions. Total RNA (1–5 *μ*g) was used for first-stand cDNA synthesis in a 20 *μ*l-reaction using 0.5 *μ*g of oligo (dT)_12–18_ (Invitrogen), 0.5 mM dNTP mix, 50 mM Tris-HCl (pH 8.3), 75 mM KCl, 3 mM MgCl_2_, 10 mM DTT, 40 units RNaseOUT recombinant ribonuclease inhibitor (Invitrogen), and 200 units of superscript II RNase H^−^ RT (Invitrogen).

PCR primers for murine IL-1*β*, IL-6, TNF, and *β*-actin genes have been described previously [26]. A 50 *μ*l PCR reaction containing 1X-SYBR Green PCR master mix (Applied Biosystems, Foster City, Calif, USA), 50 nM of each forward and reverse primers, and 2 *μ*l of the cDNA sample. Thermal cycling and data analysis were done on the ABI-prism 7700 sequence detector (Applied Biosystems). Dissociation protocol was included in the final step. The copy number of cytokine transcripts was estimated from a standard curve and the mean of cytokine mRNA levels was normalized utilizing the *β*-actin mRNA levels from each sample. The data were represented as geometric mean of fold change relative to untreated control cell cultured under identical conditions.

### 2.5. Statistic analysis

Statistical analysis was performed using the unpaired Student *t* test, two-tailed. Log transformations were performed as appropriate before the statistical analyses. Differences were considered statistically significant with *P* < .05.

## 3. RESULTS

### 3.1. Dose-dependent cytokine responses to rWSP in RAW 264.7 cells

**Figure 1:**
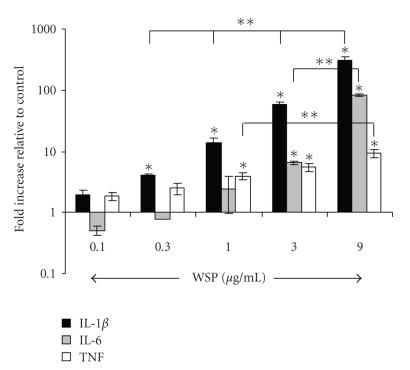
Dose response of rWSP-induced IL-1*β*, IL-6, and TNF mRNAs production in murine macrophage RAW 264.7 cells. The macrophage cells were incubated with various concentrations of rWSP for 3 hours. The data represent mean values and standard deviations of fold increase of cytokine transcripts relative to negative control in log scale. Significant differences to untreated controls (*P < .01 for IL-1*β* mRNA levels and *P* < .05 for IL-6 and TNF mRNA levels) and to rWSP-stimulated cells at 9 *μ*g/mL (**P < .05) are indicated.

At three hours postexposure, expression of IL-1*β* and IL-6 in RAW 264.7 cells in response to the rWSP was dose-dependent (Figure 1). TNF transcription appeared to be less responsive to different concentrations of rWSP used in this study. In the RAW 264.7 cells, the rWSP appeared to preferentially induce IL-1*β* over the 3-hour exposure period which ranged from a 4-fold increase at 0.3 *μ*g/mL to a ∼300-fold increase in transcription at the 9.0 *μ*g/mL. Although lower, IL-6 was also significantly elevated by the rWSP stimulation at 3 *μ*g/mL (6-fold) and 9 *μ*g/mL (82-fold). Although the transcription levels of IL-6 appeared to drop below that of the untreated controls at the two lowest concentrations of rWSP, these changes were not statistically significant. In contrast, during the 3 hours of stimulation, the change in TNF transcription, while significantly elevated compared to untreated controls, remained under 10 folds for all of the concentrations of rWSP tested.

### 3.2. Early induction of IL-1*β* mRNA expression, followed by expression of TNF and IL-6 mRNAs

**Figure 2:**
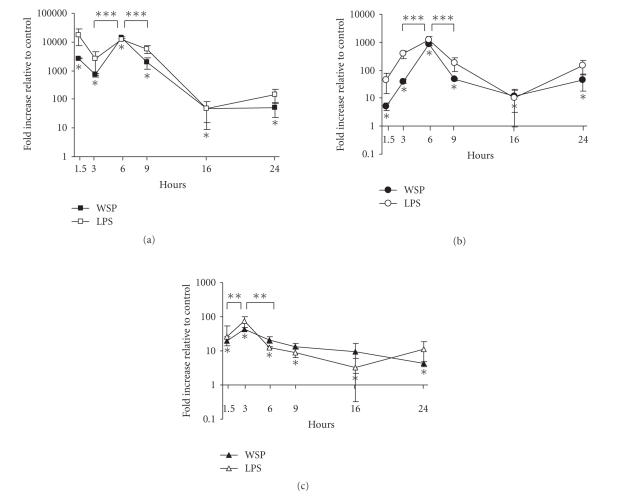
Time-course analysis of rWSP-induced IL-1â (a), IL-6 (b), and TNF (c) mRNAs expression in murine macrophage RAW 264.7 cells. Incubation of the macrophage cells with 9.0 *μ*g/mL rWSP (black symbols) or 0.1 *μ*g/mL *E. coli* LPS (white symbols) were performed at various time-points. The data represent mean values and standard deviations of fold increase of cytokine transcripts relative to negative control in log scale. Significant differences to untreated controls are indicated as *P < .05. ** Significant differences were found between rWSP-stimulated cells at 1.5 hours and 3 hours postexposure as well as between rWSP-stimulated cells at 3 hours and 6 hours postexposure (for TNF mRNA levels; *P* < .05), while *** significant differences were found between rWSP-stimulated cells at 3 hours and 6 hours postexposure as well as between rWSP-stimulated cells at 6 hours and 9 hours postexposure (for IL-1*β* and IL-6 mRNA levels; *P* < .01).

The kinetics of IL-1*β*, IL-6, and TNF gene expressions in RAW 264.7 cells was determined using the 9.0 *μ*g/mL level of rWSP at various time points (Figure 2). The rWSP induced significant increases in the transcription of all three cytokines as early as 1.5 hours postexposure with IL-1*β* showing the most robust response with over a 2500-fold increase compared to unstimulated cells. The increases at the early time point for IL-6 and TNF were more modest at 5-fold and 20-fold, respectively. The rWSP-induced transcription of IL-1*β* and IL-6 peaked between 6 and 9 hours postexposure, while the peak in TNF expression appeared to be between 3 and 6 hours. This earlier peak in TNF expression could in part explain the relatively flat responses to the different concentrations rWSP seen in Figure 1. The transcription levels of IL-6 that appeared to increase again at 24 hours postexposure were not statistically significant whereas TNF expression had dropped to near the levels of seen in the untreated control by this time. The magnitude and kinetics of the cytokine responses to 9.0 *μ*g/mL rWSP paralleled those observed in control RAW 264.7 cells exposed to 0.1 *μ*g/mL of *E. coli*-derived LPS (Figure 2).

### 3.3. The cytokine responses to rWSP were not due to LPS contamination

**Figure 3:**
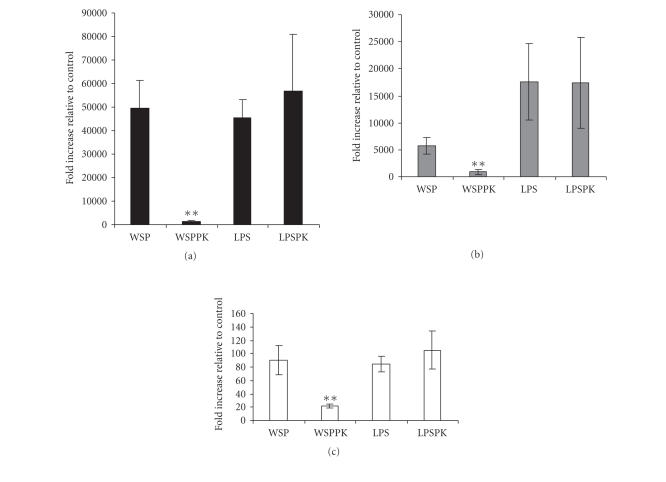
Effect of proteinase *K* treatment in rWSP-induced IL-1â (a), IL-6 (b), and TNF (c) mRNAs expression in macrophage RAW 264.7 cells. The macrophage cells were incubated with 9 *μ*g/mL rWSP (WSP), 9 *μ*g/mL rWSP pretreated with proteinase K (WSPPK), 0.1 *μ*g/mL LPS (LPS), and 0.1 *μ*g/mL LPS pretreated with proteinase K (LPSPK) for 3 hours. The data represent mean values and standard deviations of fold increase of cytokine transcripts relative to negative control. Significant differences were found between WSP- and WSPPK-treated cells (*P* < .05; indicated by **).

**Figure 4:**
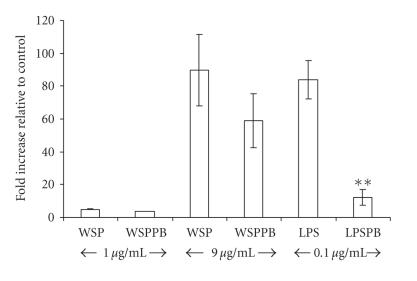
Effect of polymyxin B treatment in rWSP-induced TNF mRNA expression in macrophage RAW 264.7 cells. The macrophage cells were incubated with rWSP (WSP; 1 and 9 *μ*g/mL), polymyxin B-treated rWSP (WSPPB), LPS (LPS; 0.1 *μ*g/mL), and polymyxin B-treated LPS (LPSPB) for 3 hours. The data represent mean values and standard deviations of fold increase of cytokine transcripts relative to negative control. Significant differences were found between LPS- and LPSPB-treated cells (*P* < .01; indicated by **).

Because the rWSP used in these studies was derived from an *E. coli* extract, it was possible that all or part of the response was due to the trace contamination of bacterial LPS (0.17 EU/mL). To test this possibility, an aliquot of the rWSP preparation was pretreatment with proteinase K prior to incubation with the RAW 264.7 cells. The protease treatment of the rWSP preparation drastically abrogated the transcription of IL-1*β*, IL-6, and TNF cytokines in macrophage RAW 264.7 cells (Figure 3). The same treatment had no effect on the activity of LPS. To further test for a possible role of LPS in the rWSP response, polymyxin B, an LPS-neutralizing agent, inhibited LPS-induced TNF mRNA expression by almost 90% (reduced from 84 folds to 12 folds; *P* < .01) in the macrophage cells, but this treatment had no demonstrable effect on either low- or high-dose rWSP-induced TNF transcriptions (Figure 4). Taken together, the results of the protease digestion and polymyxin B experiments strongly indicate that the trace LPS contamination of the rWSP preparation did not significantly contribute to the ability of the rWSP preparation to induce innate cytokine transcription from RAW 264.7 cells.

## 4. DISCUSSION

The pathology of drug-induced adverse reactions in lymphatic filariasis is characterized by the increased post-treatment concentrations of proinflammatory cytokines, and inflammatory mediators [12–14]. Traditionally, these adverse reactions have been blamed on IgE-mediated responses triggered by mass release of parasite antigens—presumably from dead and dying parasites [27]. However, there are little data to support this mechanism [11]. In a murine model, significant levels of TNF and detectable adverse reactions were induced after antifilarial chemotherapy of naïve mice transfused with *B. malayi* microfilariae [23]. These results from animals that did not have time to produce an adaptive immune response suggests that at least some component of the pathology of drug-induced adverse reactions are due to innate immune responses rather than the adaptive immune response. The innate immune response can play an important role in the pathogenesis of lymphatic filariasis, since infection of immunodeficient mice with *B. pahangi* results in the development of T cell-independent lymphedema [28]. In addition, the pathology in T cell-deficient animals is associated with the accumulation of macrophages, and the local secretion of inflammatory cytokines, including IL-1â, IL-6, TNF, and GM-CSF in parasitized lymphatic vessels [28, 29].

*Wolbachia* are a key determinant to the induction of innate inflammatory responses in vitro and in vivo studies, and have been implicated to play an important role in the pathogenesis of human lymphatic filariasis [19, 20, 23, 30, 31]. The TNF production of mouse macrophages and neutrophils induced by *B. malayi* extracts is dependent on the presence of *Wolbachia* [19, 31]. While initial studies using the *Wolbachia* from *B. malayi* indicated that LPS-like molecules were major mediators of *Wolbachia*-mediated inflammatory responses, it is now clear from the results of genome sequencing that the *B. malayi Wolbachia* does not encode the enzymes required for LPS synthesis [15] implying that other *Wolbachia*-derived molecules are mediators of these powerful innate immune responses.

In this study, purified rWSP derived from *B. malayi Wolbachia* proved to be a potent activator of the innate immune system, as determined by the pronounced expression of proinflammatory cytokine genes in the murine macrophage RAW 264.7 cells. A role for WSP as an inducer of innate responses is support by the findings that a recombinant form of the WSP found on the surface of the *Wolbachia* harbored by the filarial species *Dirofilaria immitis* was capable of inducing a rapid secreting of cytokines from human PBMC [24]. Hence, WSPs from different filarial nematode hosts have a common ability to elicit proinflammatory responses in cells of innate immune system. However, it is of interest to determine whether different WSP species exhibit the same characteristic of innate inflammatory response. It is generally agreed that adverse reactions are more severe in *Brugia*-infected individuals compared to those infected with *W. bancrofti* [11]. Additional studies are necessary to determine if these differences in clinical outcome are due specifically to WSP-host cell interactions or other bacterial-derived molecules.

The transcription of proinflammatory cytokines in response to the rWSP was dose-dependent and IL-1*β* was the dominant transcription response in RAW 264.7 cells. The response to rWSP was rapid with significantly elevated IL-1*β*, IL-6, and TNF after only 1.5 hours of exposure. It will be interesting to determine if the magnitude and kinetics of the innate response documented here for murine-derived cells is recapitulated in human cells.

Studies have been conducted to determine the innate receptors that important for host interactions with *Wolbachia*-derived molecules. Activation of innate inflammatory responses by the *D. immitis Wolbachia* WSP could be affected by signaling through both TLR-2 and TLR-4 [24]. In contrast, the innate inflammatory pathways activated by extracts containing the *Wolbachia* from *B. malayi* or *Onchocerca volvulus* were shown to be dependent only on TLR2–TLR6 interactions and dependent on the adaptor molecules MyD88 and TIRAP/Mal [32]. It is likely that the rWSP used in this study also signaled through TLR2–TLR6. At this time, it is unclear what the structural relationship between the WSP from the *B. malayi Wolbachia* and the known TLR2–TLR6 ligands such as lipoproteins, peptidoglycans, lipoarabinomannans [33].

Although the role of *Wolbachia* in the pathogenesis of adverse antifilarial drug reactions has not been definitively established, the results of a recent study provide evidence to support this hypothesis. In the results of clinical studies, prior treatment of patients infected with *W. bancrofti* with a 3-week course of doxycycline to deplete *Wolbachia* prevented adverse reactions during subsequent albendazole and ivermectin treatment that resulted in worm killing. Importantly, for individuals in the group that did not receive doxycycline the levels of *Wolbachia* released into plasma were related to the incidence of adverse reactions and to the levels of plasma proinflammatory cytokines [34].

In conclusion, the WSP from the *B. malayi Wolbachia* elicited murine macrophages to rapidly upregulate the transcription of the proinflammatory cytokines IL-1*β*, IL-6, and TNF. Therefore, *Wolbachia*, through their WSP, could play a role in the initiation of inflammatory responses in human patients that are associated with antifilarial drug treatment. The characteristics and mechanisms of rWSP-induced IL-1*β*, IL-6, and TNF responses would be valuable knowledge for alternative prevention and treatment of the drug-induced adverse reactions.
